# Challenges in the Treatment of Low-risk Gestational Trophoblastic Neoplasia

**DOI:** 10.1055/s-0041-1735177

**Published:** 2021-08-30

**Authors:** Antonio Braga, Kevin M. Elias, Neil S. Horowitz, Ross S. Berkowitz

**Affiliations:** 1 Department of Obstetrics and Gynecology, Centro de Doenças Trofoblásticas do Rio de Janeiro, Universidade Federal do Rio de Janeiro, Rio de Janeiro, RJ, Brazil; 2 Department of Maternal Child, Universidade Federal Fluminense, Niterói, RJ, Brazil; 3 Division of Gynecologic Oncology, Department of Obstetrics, Gynecology and Reproductive Biology, New England Trophoblastic Disease Centre, Brigham and Women's Hospital, Dana-Farber Cancer Institute, Harvard Medical School, Boston, MA, United States

Gestational trophoblastic neoplasia (GTN) is a rare tumor that arises from placental tissues and exhibits a high cure rate when treated with cytotoxic chemotherapy. 1 Although its most common origin is hydatidiform mole, GTN can develop from any type of pregnancy: abortion, ectopic pregnancy, or preterm/term gestation. 1

The early diagnosis of GTN is the key to ensure cure as patients with late diagnosis often have metastatic disease and require more aggressive and toxic treatment and experience a worse prognosis. 2 While GTN is highly sensitive to chemotherapy, it is important to differentiate which patients will respond to single-agent chemotherapy versus those that will require more morbid, multiagent regimens to achieve remission. 3 4 To this end, the World Health Organization (WHO) and the International Federation of Gynecology and Obstetrics (FIGO) created a combined anatomical staging and clinical prognostic risk scoring system which identifies patients with a higher risk of resistance to single-agent chemotherapy. 5 Patients with a WHO/FIGO risk score ≤ 6 are considered to have low-risk GTN and are treated with single-agent chemotherapy while those with a score ≥ 7 are classified as having high-risk GTN and are treated with multiagent chemotherapy. 5

In this scenario, there are 5 important challenges for the treatment of low-risk GTN, which represent about 80% of these tumors: proper assignment of FIGO prognostic score; treatment of first-line single-agent chemoresistant GTN; regimen of choice to treat GTN with FIGO score of 5 or 6; alternatives for precision treatment of low-risk GTN and the best strategy to reduce the lethality of these tumors. The purpose of the current editorial is to present a guide to good practices that can address these issues.

Since GTN treatment is directly related to the FIGO prognostic score, 5 correct assessment of score is essential for the appropriate selection of chemotherapy. The prognostic factors involved in this classification are maternal age, gestation index, interval between the end of antecedent gestation and the beginning of chemotherapy, pretreatment serum hCG level, largest tumor size (including uterus), site, and number of metastases and previous failed chemotherapy treatments. 5

The most common site of GTN metastasis is the lung, 6 7 and, as such, the size and number of lung metastases is fundamental for a correct assessment of the WHO/FIGO prognostic score. Although FIGO expressly recommends using chest X-ray for screening for GTN lung metastases, 5 8 the use of chest computed tomography (CT) has become increasingly common in cancer staging, not only because of its higher sensitivity for detecting metastatic nodules, but also for the more accurate measurement of tumor size. Although CT improves prediction of single-agent chemotherapy resistance, it does not influence overall treatment outcome or the time to hCG normalization. 7 On the contrary, because it has higher sensitivity in detecting micrometastases, which may be seen in ∼ 40% of patients, chest CT can distort the FIGO score, leading patients with low-risk GTN, who would be largely cured with single-agent regimens, to receive multiagent chemotherapy. 9

Thus, we emphasize the importance of basing the scoring of metastases according to the FIGO recommendations, which consists of a pelvic exam to assess genital metastases, Doppler pelvic ultrasound, and chest X-ray. 10 If the chest x-ray is normal, screening can be stopped. Only in cases of lung lesions larger than 1 cm or when there are doubts about the presence of pulmonary metastases should patients be subjected to more detailed imaging studies (such as chest computed tomography and magnetic resonance imaging of the brain and abdomen). 10

For patients with low-risk GTN, the treatment of choice in Rio de Janeiro and Boston is an 8-day regimen of methotrexate (1 mg/kg intramuscularly days 1, 3, 5 and 7) with folinic acid rescue (15 mg fixed dose per os days 2, 4, 6, and 8) (MTX/FA). 11 12 13 14 In cases of chemoresistance, the preferred regimen of choice is pulsed actinomycin-D (Act-D) (1,25 mg/m2 intravenous push), especially in cases in which the hCG level is below 1,000 IU/L. 15 Patients with levels above this cutoff may benefit from multiagent chemotherapy containing etoposide, MTX, Act-D, cyclophosphamide, and vincristine (EMA-CO multiagent regimen). 15

Unfortunately, since 2013, Brazil has experienced periodic shortages of Act-D, compromising the treatment of GTN in patients with MTX/FA resistance. 16 Without access to Act-D, many GTN reference centers (RCs) in Brazil have been using intravenous carboplatin (target area under the curve of 6) every 3 weeks to treat women with MTX/FA chemoresistance. 16

Despite the fact that a Sheffield study evaluating patients with MTX chemoresistance treated with carboplatin showed a remission rate of 80.9% (17/21), 17 Brazilian data found much lower response rates (47.8%; 11/23), with a higher occurrence of hematological toxicity, notably anemia (30.4%), lymphopenia (47.7%), and thrombocytopenia (43.4%), as well as a higher occurrence of febrile neutropenia (14.4%) and vomiting (60%). 16

These results demonstrate the importance Act-D and the critical role of government dialogue to regulate the availability of this important orphan drug for this not so rare disease in Brazil.

Despite the widespread adoption of the WHO/FIGO Prognostic Scoring System (FIGO 2002), concerns have been raised regarding the subgroup of patients with low-risk GTN, with FIGO risk score of 5 or 6. The clinical remission rate for these patients, treated with single-agent chemotherapy, only reaches 31 to 35%, which contrasts sharply with the best results observed in cases of FIGO risk scores of 0 to 4, in which remission rates are 60 to 65%. 18 It remains controversial whether patients with a FIGO score of 5 or 6 should be treated initially with single or multiagent chemotherapy. 3 19 Some authors advocate that treatment with single-agent chemotherapy in this population should be avoided because, in addition to delaying the time to remission, this might contribute to chemoresistance. 3 18 Conversely, others have argued that, since these patients ultimately achieve a high cure rate approaching 100%, even when resistance to single-agent chemotherapy develops, it is reasonable to treat these patients initially with the less toxic single-agent therapy in hopes of avoiding multiagent regimens. 20 21 22

An international collaborative study (London, Rio de Janeiro, and Boston) of the largest world data set of FIGO 5/6 GTN patients noted that approximately 60% of women with FIGO 5/6 GTN achieve remission with single-agent chemotherapies used initially or sequentially. 23 The rest are nearly all cured with subsequent multiagent treatment. 23 The use of single-agent chemotherapy for these patients avoids exposure not only to immediate side-effects like alopecia and myelosuppression, but also long-term sequelae including earlier menopause and increased future risk of leukemia. 3 4

The important finding of this study was the identification of prognostic factors that effectively guide the treatment of patients with GTN and FIGO score of 5 or 6. Amongst patients with no metastases and no choriocarcinoma with hCG < 411,000 IU/L, single-agent chemotherapy has a positive predictive value (PPV) of 80% to achieve remission. Amongst patients with either metastases or choriocarcinoma and an hCG < 149,000 IU/L, single-agent chemotherapy, again, achieved remission in 80%. For patients with metastatic choriocarcinoma, regardless of hCG level, multiagent chemotherapy should be promptly initiated since no patient achieved remission with single-agent chemotherapy. 23

Precision treatment has arrived in GTN through immunotherapy. Programmed cell death ligand 1 (PD-L1) and its programmed cell death protein 1 (PD-1) receptor are strongly expressed by GTN, suggesting the ligand is involved in tumor-immune evasion. The French group presented the results of treatment with avelumab, an anti-PD-L1 human monoclonal antibody (10 mg/kg intravenously every 2 weeks) for patients with low-risk GTN and chemoresistance to single-agent chemotherapy. 24 Eight of 15 patients (53.3%) achieved remission after a median of 9 avelumab cycles, with minimal early toxicity and no relapse after 29 months of follow-up. 24 Importantly, they did report a pregnancy posttreatment with avelumab. 24

Although avelumab represents a new therapeutic option in patients with low-risk GTN with chemoresistance to a single-agent regimen, the high costs of the treatment and lower remission rate, when compared to second-line Act-D, mean that this treatment still has little clinical role in low-risk GTN. 25 Currently, the French Trophoblastic Group is recruiting patients to assess the performance of avelumab plus MTX for first-line low-risk GTN treatment. 26

Although GTN is a highly curable disease, especially in low-risk cases, women still die from this disease. A Brazilian study evaluating 2,186 patients with GTN observed that patients with low-risk disease had a significantly higher risk of death if they had choriocarcinoma (relative risk–[RR]: 12.40), metastatic disease (RR: 12.57), chemoresistance (RR: 3.18), or initial treatment outside a RC (RR: 12.22). 27

The setting of treatment has a profound impact on the outcome of this disease and on the occurrence of death due to GTN. The best strategy for reducing death from GTN is to treat these patients in a RC, the only modifiable risk factor associated with death due to GTN. 2 The Brazilian experience clearly shows that when these patients are followed in a RC, they have a lower metastasis rate and shorter median time interval between molar evacuation and chemotherapy onset than those initially treated outside the RC. 27

Between advances and challenges, the truth is that GTN is still a relatively unknown disease for many physicians in the world. The scientific dissemination of information about this highly curable disease should draw physicians' attention to clinical suspicion and immediate referral to specialized services. The best chance for a GTN patient to be cured is the highest quality of treatment beginning at the onset of her illness, which can be best achieved in a RC.
